# HTS-DB: an online resource to publish and query data from functional genomics high-throughput siRNA screening projects

**DOI:** 10.1093/database/bat072

**Published:** 2013-10-10

**Authors:** Rebecca E. Saunders, Rachael Instrell, Rossella Rispoli, Ming Jiang, Michael Howell

**Affiliations:** High-throughput Screening Laboratory, Cancer Research UK, London Research Institute, 44 Lincoln's Inn Fields, London WC2A 3LY, UK

## Abstract

High-throughput screening (HTS) uses technologies such as RNA interference to generate loss-of-function phenotypes on a genomic scale. As these technologies become more popular, many research institutes have established core facilities of expertise to deal with the challenges of large-scale HTS experiments. As the efforts of core facility screening projects come to fruition, focus has shifted towards managing the results of these experiments and making them available in a useful format that can be further mined for phenotypic discovery. The HTS-DB database provides a public view of data from screening projects undertaken by the HTS core facility at the CRUK London Research Institute. All projects and screens are described with comprehensive assay protocols, and datasets are provided with complete descriptions of analysis techniques. This format allows users to browse and search data from large-scale studies in an informative and intuitive way. It also provides a repository for additional measurements obtained from screens that were not the focus of the project, such as cell viability, and groups these data so that it can provide a gene-centric summary across several different cell lines and conditions. All datasets from our screens that can be made available can be viewed interactively and mined for further hit lists. We believe that in this format, the database provides researchers with rapid access to results of large-scale experiments that might facilitate their understanding of genes/compounds identified in their own research.

Database URL: http://hts.cancerresearchuk.org/db/public

## Introduction

RNA interference (RNAi) is a natural mechanism for gene silencing that is now widely used in large-scale loss-of-function screening campaigns in the form of small-interfering RNA (siRNA) libraries that can be used to knock down genes in a high-throughput manner. Although the experimental process of using RNAi is relatively simple, it is by no means trivial to physically manipulate the large number of plates involved, with challenges also arising from the need to track and analyse the millions of data points such screens can generate. To overcome these challenges and make genome-wide RNAi screening accessible to diverse research groups, many research institutes have established core–high-throughput screening (HTS) facilities that can use and perfect the specialist machinery, protocols and analysis required to operate efficiently on such a large scale.

Such core screening facilities may work on straightforward screening projects with routine assays or readouts, or, as in our case, may be required to conduct many diverse projects simultaneously using a wide variety of assays and cell lines. In addition to biological complexity, our projects can consist of a single screen in one cell line using a single library, or, as is more often the case, may include several different screening stages. For example, an initial pilot screen using a small library of selected siRNA reagents could be followed by several genome-wide screens in different cell lines or assay conditions and finally concluded using follow-up secondary screens, to repeat and validate putative hits by deconvolution of the original pooled siRNA screening reagents (1). Furthermore, each individual screen may include several measurements, some of which may be required for hit selection within the project (e.g. intensity of an antibody stain), and others that can be supplementary to the project (e.g. cell number, nuclear morphology) but nonetheless of some biological relevance. High-content screens provide an additional level of complexity, where image analysis algorithms produce many measures reflecting different, but overlapping, aspects of the underlying biology (e.g. spot count, spot intensity and spot area). Because of these complexities, our facility requires a complex informatics solution to manage projects and reagent libraries and within these projects link data from secondary and downstream work to data from initial screening studies.

Simply recording data is, however, not sufficient on its own, as users must also be able to interact easily with their data to assist in interpreting the outcome of screens. For our facility, it is important to make our screening data available within the institute to engender scientific collaboration and make the screening data applicable to research groups with diverse interests. On publication of our projects, it also becomes important to share our data to the wider research community (2). There are several excellent online repositories of genome-wide and smaller scale siRNA screening data such as Genome RNAi (http://genomernai.de/GenomeRNAi/), ROCK (http://rock.icr.ac.uk/), FLIGHT (http://flight.icr.ac.uk/) and Pubchem (http://pubchem.ncbi.nlm.nih.gov/). These repositories often provide the data within a gene-centric view, allowing diverse research groups to find out whether a wide variety of screens have identified a gene of interest as a hit or not. However, it is often hard to judge the significance of an individual hit within context of the screen, and the users often have to rely on the original screener’s definition of hit and non-hit.

Genome-wide siRNA data can be useful to not only understand the underlying biological process covered by the original screen and genes of interest, but also for meta-analysis on a wide variety of biological processes across different cell lines. Such meta-analysis can mine screen data from several projects to identify and cluster groups of genes that act similarly within different groups of cell lines or conditions. To make screening data applicable for meta-analysis, it is important to describe thoroughly the experimental techniques and readouts, as well as inform about the analysis techniques applied to normalize and score data. In the past, several different analysis techniques have been developed and applied to RNAi screening data to control quality and identify hits, where different methods are applied based on inherent qualities of the data (3). Comparison of two independent genome-wide siRNA screens for host factors of yellow fever virus infection identified that reproducibility was significantly influenced by the analysis methodology chosen (4). Because of these complexities, when reporting HTS data, it is important to detail which analysis methods have been used and in addition provide raw un-normalized data if appropriate.

The HTS-DB described here has been created in-house from scratch and evolved over a number of years of use to meet all of the demands of genome-wide siRNA screening in a busy core facility. It provides full data tracking, interactive data visualization and analysis and comparison of screening results both within projects and between different projects. Here we describe the public open-access view of our system, which allows us to disseminate fully annotated screening data from our published projects. It also gives us the opportunity to immediately make available the additional genome-wide datasets relating to cell viability that are not the primary focus of individual projects, and so do not have to wait until publication of the screen before being made available.

### The HTS-DB

Our database system is a web-based laboratory information management system to track and store our projects, screens, assays, cell lines and library information. The design allows us to track each individual plate and library stock through all stages of an assay and project.

**Figure 1.**
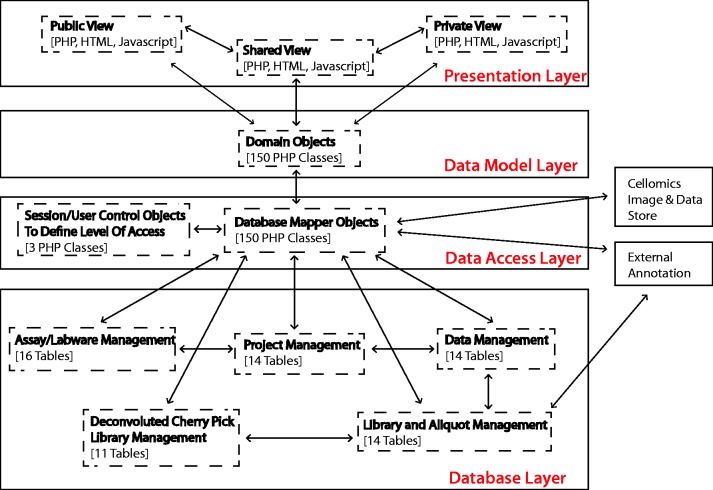
Summary of system architecture and organization of the HTS-DB (as outlined in the text).

To provide a long-term, scalable and flexible solution, the system architecture is currently built using PHP v5.1.5 and MySQL v5.0.77, and applies concepts of object-orientated programming to decouple the logical database layer, data access layer and presentation layer (Figure 1). Dynamic client-side functionality is implemented with Javascript and AJAX (Asynchronous Javascript and XML) based on the jQuery framework. jQuery plug-ins were used where appropriate, and, in particular, the graphing functionality uses the Highcharts jQuery plugin.

The flexible multilayer structure allows for continuing evolution of the system, allowing us to change the underlying database structure, without having an impact on how the data are viewed and used in the HTML web pages and vice versa. For example, this design decision supported the integration of the private/public view, where only the data access layer is concerned with security and the access of private data, whereas only the presentation layer is concerned with the different public and private views. In addition, it can also provide flexible integration of additional library types and technologies. Within the design, there is only the requirement that a reagent has a position that can be defined on a plate. The reagent type can currently be siRNA, double-stranded RNA, microRNA, chemical or yeast deletion strain, but additional types of reagent such as short hairpin RNA (shRNA) or any other arrayed biological reagent can easily be incorporated, only requiring the addition of another database table, a domain object and a domain mapper class, without affecting any of the programming related to how users view different libraries. This modular design of libraries means that annotations can be managed using link tables, and additional levels of annotation can be added, when required, by mapping annotation IDs to each other. For example, although each siRNA is associated with an original intended RefSeq and gene symbol annotation, we have mapped each siRNA sequence to its current genome position within the Hg19 reference genome using Bowtie (5). Unfortunately, owing to third-party licensing issues, we cannot publish siRNA sequences or exact genome position; however, we can show how Ensembl gene IDs and Ensembl transcript IDs map to individual siRNA oligos positions using UCSC genome browser reference tables (6). In a similar manner, the individual siRNA sequences can also be mapped to current RefSeq gene annotations. These positions can be summarized for pools of oligos so that pools, as well as individual sequences, can be classified based on whether the sequences map to the genome as expected. The PHP annotation mappers are flexible so that additional functions can be added to retrieve additional annotation. Currently, the database links library compounds to GenomeRNAi, NCBI NuCore, NCBI Probe, NCBI PubChem Substance, Ensembl, RefSeq, Flybase and Flight databases where appropriate.

The complexity of screen multiparametric data and normalization techniques also represented a design challenge. Two levels of data objects are used where raw and normalized data are treated differently. With raw data, a data point is stored for each well, within each plate, within each replicate, so that replicate information is available and effect size can be estimated. Normalized data are stored as a summary score of the replicates so that each pool can be represented by one value within the screen and dataset. Each normalized dataset has a raw source dataset, but the mapper hierarchy is flexible, so that analysed datasets built from other analysed datasets (e.g. where the differential of control and drug screens is calculated) can also be included. Within the analysed datasets, both the rank and normalized scores are included so that datasets can be rapidly evaluated in our search functions without the need to access the data for the whole screen to calculate a rank.

## Database tools

Our database provides several different tools to search and browse screening data. Principally, it is designed to serve two main purposes: (i) provide all information and data for a chosen screen or project and (ii) provide information from all screens for a chosen gene.

### Project level view

**Figure 2.**
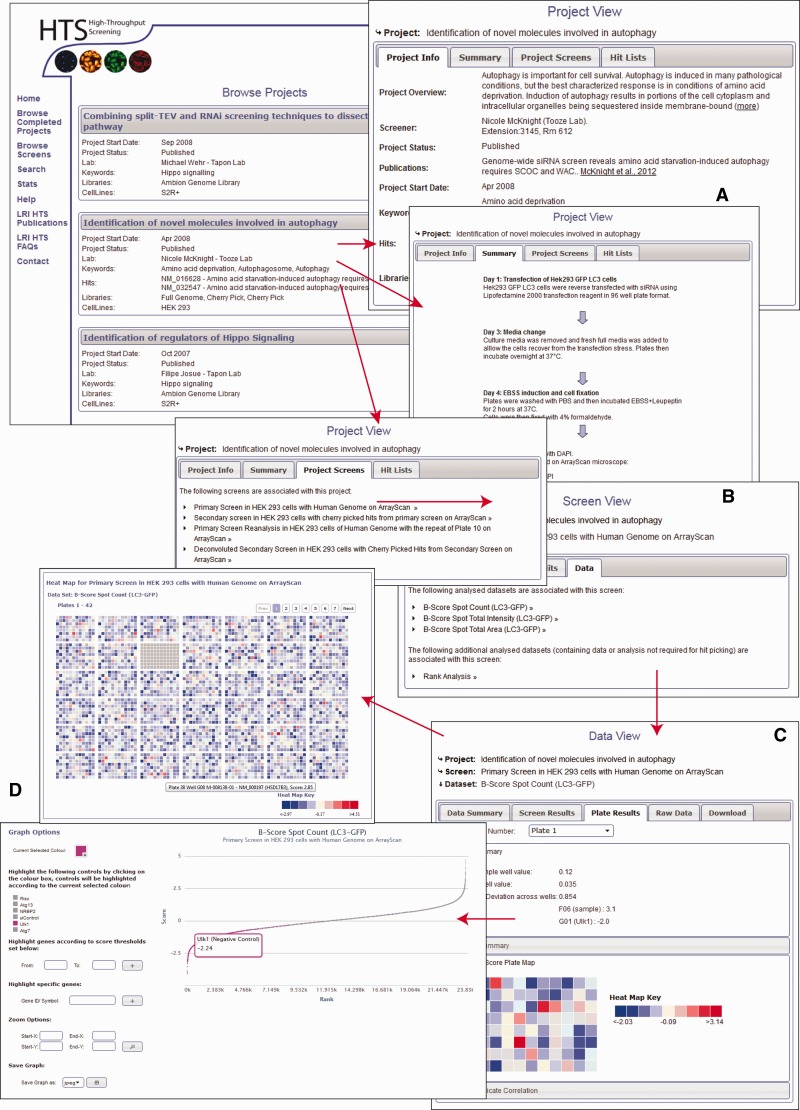
(**A–D**). Information layers of HTS-DB. Several views are shown, and the direction of arrow indicates the flow of page navigation the user can follow to navigate through a project from a description of the initial aims of the project to the raw data as described in the text.

If users are interested in a particular project, the data from that project can be viewed and studied by browsing through different levels of the database (Figure 2). The top level provides a project overview including the broad aims and intention of the project and broad descriptions of the methodology (Figure 2A). The second level focuses on the overview of the individual screens of the project and includes more detailed methodology (Figure 2B). Because most of our tools allow the user to interpret the data, rather than fixating on whether something is identified as a hit or not, based on an arbitrary threshold, it is imperative that we provide all the information necessary to make a valid conclusion about the scores within the screen. Each project and screen is summarized as a flow chart, but the database also includes a comprehensive assay description using a standard template based on recommendation by MIARE (Minimum Information About an RNAi Experiment) (http://miare.sourceforge.net/HomePage). The third level views the different datasets of the screen within the project and allows the user to view data, both analysed and raw, in the context of a screen, plate or an individual well (Figure 2C). Within a browsing session, users may use several graph tools to display the data for the whole screen, and interactively highlight certain genes or controls to understand the dataset more thoroughly (Figure 2D). The database also provides heatmap tools to highlight the position of scores on individual plates and colour plates by replicate standard deviation. These graphing and heatmap tools are provided so that the user can assess the quality of the data and identify possible screening artefacts such as positional or batch effects. In addition, all of the assay controls used in the screen are described and included within the database, and the distribution of controls throughout the screen can be assessed. All data can be downloaded as an XML document, fully compatible with Microsoft Excel. Extensive ‘help’ and walkthrough documentation is also provided on the site.

**Figure 3.**
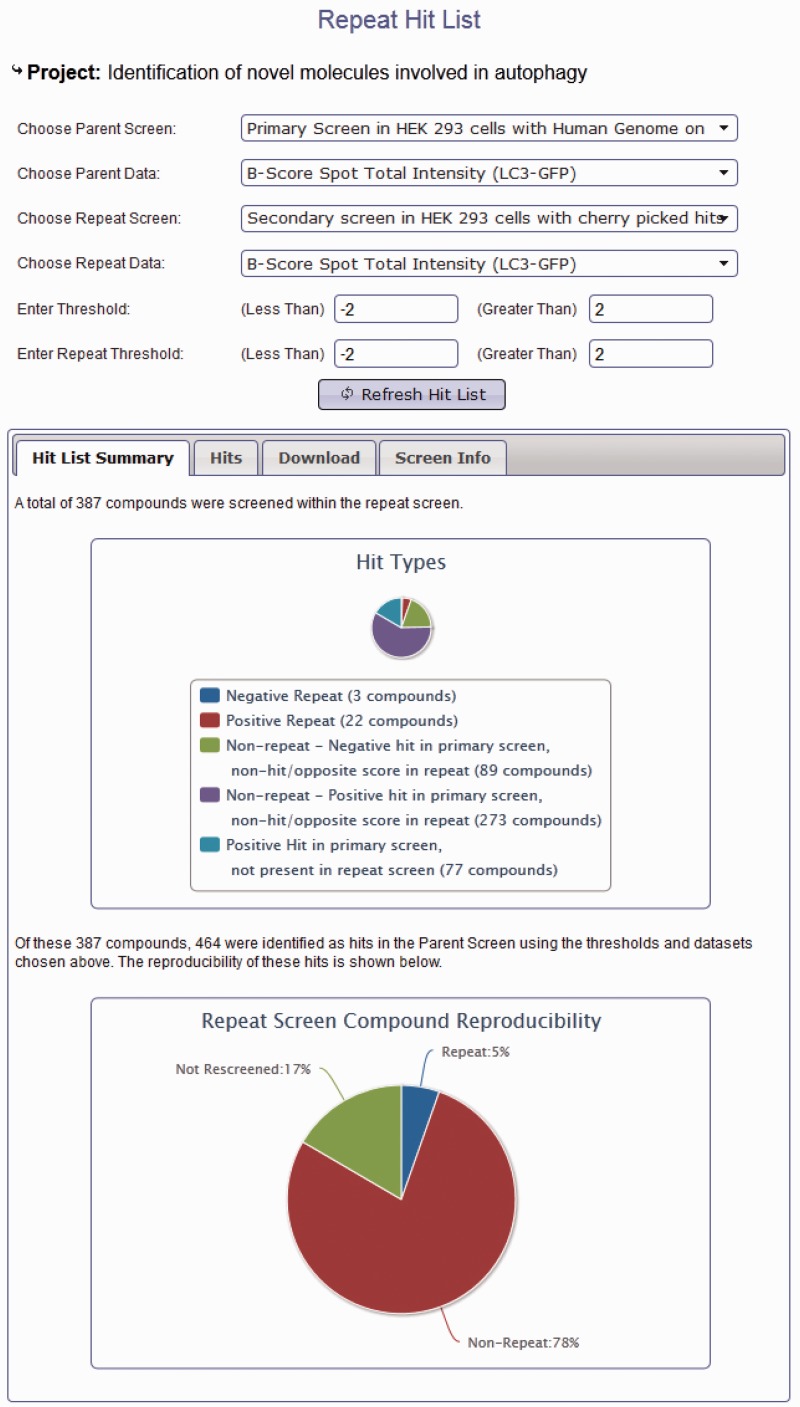
Project hit list generation using the HTS-DB. Screen shot of the hit list tool to enable users to identify repeating and non-repeating hits between primary and secondary screens as defined by their own thresholds (see text for details).

In addition to browsing different datasets within a project, data from screens within the same project can be compared using the hit list generation tools. Here, secondary or repeat screens and deconvolution screens can be compared with their primary datasets to identify overlap (Figure 3). Hit lists can be generated by interactive user-defined thresholds for any dataset within a project. Hit lists generated by the user can be summarized to show the distribution of hits across plates and wells, again in order to provide the user with some indication of the quality of their chosen datasets. All generated hit lists can also be downloaded as an XML document, fully compatible with Microsoft Excel. As our database can link secondary data to primary screen scores, this offers an invaluable resource of data that can be used to identify possible off-target effects (OTEs). For example, if all four oligos score as the primary pooled screen, it is more likely that the pooled hit is targeting the same gene. For each published project, we can provide all of the deconvoluted scores for each of our primary hits. These data are often missed in other online data repositories and publications; however, it could prove very useful for assessing likely OTEs, and in addition, could provide valuable datasets to develop possible methods for predicting the probability that a pool might have OTEs. We also provide updated Ensembl and RefSeq annotation for all our pooled library sequences so that our data can be viewed in context of current genome annotations where Ensembl transcript coverage and multiple match sites can be considered. In addition to using our data to understand possible OTEs, as we provide all our secondary data, these datasets could prove useful for calculating false-positive and false-negative rates based on different statistical analysis techniques. Note that the design of the database can also allow for the incorporation of large-scale primary screens performed with single oligonucleotides in a similar manner to those conducted using pooled siRNAs.

### Gene or reagent level view

**Figure 4.**
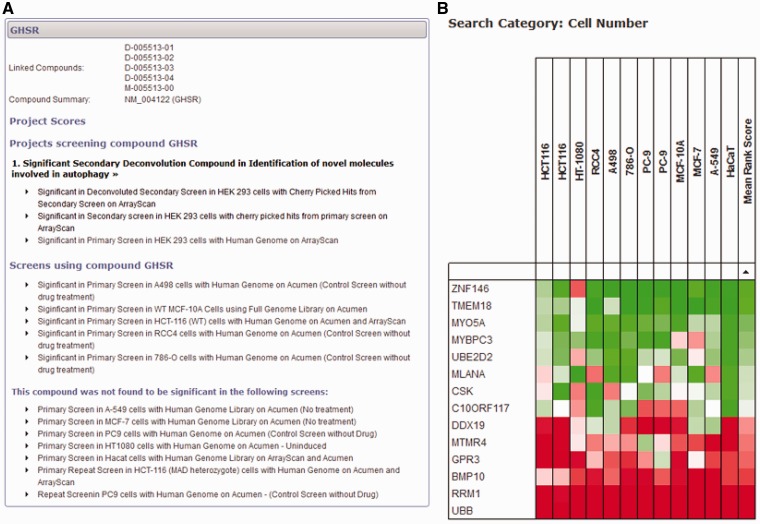
Gene-centred query of the HTS-DB. (**A**) A detailed query of the HTS-DB showing all details available for a particular gene, *GHSR*. This example shows the siRNA targeted against this gene was a significant hit in an Autophagy project, and was successfully taken forward to the secondary deconvolution stage within this project. It also lists additional screens in other cell lines where the gene scored significantly. These lists can be extended to show detail of the originating dataset for more information. (**B**) Example output from the HTS-DB Batch Query mode. This example shows the cell viability scores across 12 genome-wide screens for siRNA reagents targeting 14 genes. The effect on cell viability of siRNA knockdown of a number of genes across different screens and cell lines can be readily assessed, where, in this case, several genes show consistently low viability scores across multiple cell lines (RRM1, UBB), several show consistently high viability scores across multiple cell lines (TMEM18, ZNF146) and some show variability in their scores (CR10ORF117).

If a user is less concerned with a specific project and would like to query the database with a more gene-centric view, two different search tools can provide information across all projects for individual genes. First, a detailed gene-specific query allows the user to identify all scores within the database for all projects and datasets (Figure 4A). This view groups together individual projects, and ranks them in order of significance of the gene, allowing the user to quickly identify whether the gene of interest has been identified as significant in any project. All scores within the project can be displayed so that the user can see the results of any secondary analysis of the gene and make an informed decision about its significance as a primary hit. Within the database, we have avoided labelling library compounds and the genes they target as ‘hits’, and focus on the significance of the score within the dataset. We do this because the definition of a ‘hit’ is project-specific and possibly made up of a combination of several different measures. The fact that an siRNA reagent elicits a significant biological response in one dataset is independent of arbitrary definitions of a ‘hit' and could be of importance when viewed in other contexts.

Second, a batch query of up to 600 genes can be used to interrogate categories of data from different screens to provide a summary of each gene over the large-scale screening projects within the database. For example, several of our screens have a measure for cell viability [usually counting nuclei per well using either the Acumen Explorer cytometer (TTPLabtech) or ArrayScan microscope (Cellomics)]. In this initial release, we have only included cell viability data within this tool; however, once we are in a position to make additional common categories of data available (e.g. nuclear morphology or measures of DNA damage), the database will also be able to provide meta-analysis of these datasets. Within a batch search using cell viability data, each large-scale screen (genome-wide or druggable library) is identified, and the score based on the rank for that gene within each screen is displayed. If more than one analysed dataset is available per screen for cell viability, the most significant score is displayed. For example, if cell viability is represented by a cell count measurement from the Acumen Explorer as well as an object count using image analysis procedures from the ArrayScan, the most significant score for each gene is displayed based on the rank of that gene within each dataset. In this way, we can show a rank-based score for each gene within each screen, so that scores can be directly compared, without considering the effect size or being concerned with scores on different scales using different analysis techniques. This allows us to provide a heat map so that groups of genes can be rapidly assessed as to whether they show similar or unique patterns across screens in different cell lines or conditions. Figure 4B highlights the use of this tool for 14 genes. For example, siRNA reagents targeting UBB and RRM1 show consistent low ranks across all cell lines, indicating that knockdown of these genes is frequently determined to have a statistically significant effect on reducing cell number in several cell lines, and, therefore, that these genes are functionally required for cell viability, whereas others, such as the reagent targeting DDX19, have a more restricted effect in only some cell lines.

### Meta-analysis of cell viability data

The cell viability datasets highlighted above provide an example of the meta-analysis possible with data from a curated database. The HTS-DB currently holds 13 human cell line genome-wide screens and 2 drosophila genome-wide screens. Twelve of the 13 human genome-wide screens have cell viability data available, and this includes two screens of lung-cancer PC9 cell lines, three screens of kidney cancer lines (RCC4, A498 and 786-O lines) and two screens of breast cell lines (MCF-10A and MCF-7). To rank genes in terms of their knockdown effect across all genome-wide screens in our database, we used the redundant-siRNA activity measure (7). This method was used to rank siRNA pools from the entire genome collection according to normalized experimental effect on cell viability (Supplementary Table S1). A *P*-value for each single pool based on whether the scores for that pool are distributed significantly higher in the rankings than would be expected by chance could then be calculated. Users can download the individual datasets to conduct their own such analysis if they desire. These accumulated cell viability data are useful when hit calling, as it provides a landscape of frequently occurring activities against which we can compare every new dataset. For example, several of our screens have attempted to identify genes that when knocked down have a differential effect in two cell lines or conditions. Information from our meta-analysis has proved useful to classify those hits that are frequently involved in loss of cell viability as opposed to those hits where loss of viability is more significant for the experimental set-up and cell line. It has also been useful to highlight unusual differences in phenotypes between groups of cell lines or conditions.

**Figure 5.**
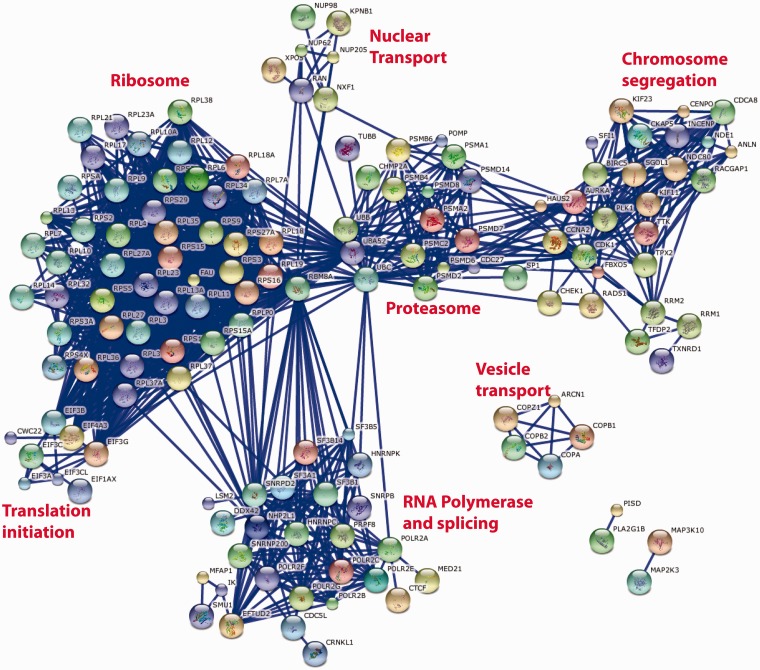
String database representation of interactions between the genes that are most commonly associated with loss of cell viability when knocked down in 12 genome-wide screens of 11 different cell lines. The top 250 genes from Supplementary Table S1 were used in the analysis. We have included all of the possible gene symbol matches to each siRNA pool when the oligonucleotide sequences of the individual siRNAs are mapped to the reference genome. The data were analysed on the STRING http://string-db.org/database using all of the default active prediction methods and displaying only the highest confidence interactions (edges). Only connected genes are shown. Clusters of functionally related and interacting genes are indicated.

Such cross-screen analysis can be taken further. Figure 5 shows the most significant interactions [as defined by the STRING database (8)] between the top 250 genes identified as having a consistent and significant effect on cell viability when knocked down with the appropriate siRNA pool. The prominent clusters identified by this analysis correspond to large multi-protein complexes required for fundamental biological processes that might be expected to have an impact on cell viability such as the ribosome, proteasome, RNA splicing and RNA Polymerase II transcription, chromosome separation and coated vesicle transport. It is interesting to note that such an analysis does not identify any conserved growth factor signalling pathways, perhaps reflecting the cell-specific nature of the growth positive pathways or their redundant nature within any single cell type. It is also noteworthy that not all recognized components of the known multi-protein complexes are identified (e.g. in the case of the proteasome) and that not all known multi-protein complexes (9) are identified, e.g. DNA replication initiation and elongation, Arp2-3 complexes. While this could reflect the ability of the individual genes of these complexes to be susceptible to siRNA knockdown, similar analyses of other common datasets (e.g. nuclear morphology or DNA damage) suggest that this is not the case and that it is simply that knock down of these genes does not necessarily reveal itself as a viability phenotype (data not shown). Quite why cell viability is robust to siRNA knockdown of the replication machinery is an interesting yet open biological question.

## Conclusion and future perspectives

The management of high-throughput functional genomics data is complex. As well as maintaining records of complex libraries with ever-changing annotation, data must also be stored so that it can be queried and mined for biological meaning. The HTS-DB system describes all assays and projects, links secondary data together with primary screen information and refrains from classifying hits based on arbitrary thresholds defined by the aim of the project. This allows the user to interpret and analyse the data based on their own interests. Although other excellent database tools have been developed, we believe our database provides a novel resource of information for high-throughput RNAi screening projects.

Our high-content screen data are managed by third-party software (Cellomics). Using their proprietary application programming interface (API), we have designed mappers to link our MySQL database to the high-content data our microscope produces. This means we can show images alongside their associated raw and normalized multi-parametric data points. This gives us an excellent tool to share image data across our institute without the need for third-party software and licences. Although we currently do not yet have high-content screens available for the public view of the database, it means that in future we can provide access to our images from screens so that phenotypes can be interpreted visually as well as using the associated normalized score.

Currently, our core facility is expanding into additional functional genomics tools such as shRNA screening, and as newer technologies become available, the need for a flexible yet cohesive database system is paramount. Our system is designed to incorporate different types of library compounds (such as well-characterized small molecule modulators, shRNA, microRNA and yeast knockout strains) so that we can readily adapt tools to display additional functional genomics screening data. As data can be provided with a gene-centric view, this will allow users to view results of several different types of screens for each gene, with different technologies providing a much more rounded view of the function of a gene of interest. This not only facilitates information sharing between members of our own facility, it may also provide a basis for future international collaborations within the wider research community.

If other core facilities would like to implement our solution or would like to deposit their data in this database, we would encourage them to contact us.

## Supplementary Data

Supplementary data are available at *Database* online.

Supplementary Data
