# Supplementary material for: HTS-DB: an online resource to publish and query data from functional genomics high-throughput siRNA screening projects
Source: Database (Oxford). 2013 Oct 10;2013:bat072. doi: 10.1093/database/bat072 (PMC3796064; doi:10.1093/database/bat072)
Supplement: Supplementary Data [file supp_2013_bat072_index.html]

HTS-DB: an online resource to publish and query data from functional genomics high-throughput siRNA screening projects — Supplementary Data 

# HTS-DB: an online resource to publish and query data from functional genomics high-throughput siRNA screening projects

## Supplementary Data

files

**Files in this Data Supplement:**

- Supplementary Data - xlsx file
